# 一个表现为血栓的遗传性低异常纤维蛋白原血症家系

**DOI:** 10.3760/cma.j.issn.0253-2727.2022.10.012

**Published:** 2022-10

**Authors:** 冲 魏, 昊 蔡, 宝来 华, 铁楠 朱

**Affiliations:** 中国医学科学院、北京协和医学院北京协和医院血液内科，北京 100730 Department of Hematology, Peking Union Medical College Hospital, Chinese Academy of Medical Sciences & Peking Union Medical College, Beijing 100730, China

纤维蛋白原（Fbg）由肝脏合成及分泌，是血浆中浓度最高的凝血因子。纤维蛋白原是由三对多肽链（Aα、Bβ、γ）以二硫键连接而成的二聚体，三条肽链分别由位于4号染色体长臂的FGA、FGB和FGG三个基因编码[1]。遗传性纤维蛋白原异常作为一种罕见的遗传性出血性疾病主要包括两种类型，一类为纤维蛋白原量的减少或缺乏，包括遗传性低纤维蛋白原血症和无纤维蛋白原血症。另一类则为纤维蛋白原的缺陷，包括遗传性异常纤维蛋白原血症（CD）和遗传性低异常纤维蛋白原血症，其中前者以纤维蛋白原活性（Fbg∶C）降低而抗原（Fbg∶Ag）水平正常为特征，后者纤维蛋白原活性和抗原均有下降但活性降低更为明显。CD和遗传性低异常纤维蛋白原血症患者在临床上既可能表现为出血，亦可能发生有血栓事件，甚至在同一患者上述两种表现共存[1]–[2]。该类疾病患者的临床表现通常难以预测，目前仅有少数基因型与临床表型存在明确对应关系，因此对这些患者的管理极具挑战。本文中我们对一个首次报道的遗传性低异常纤维蛋白原血症家系的基因型及患者表型进行介绍。

## 对象与方法

1. 家系资料：先证者为女性，32岁，2009年（26岁）产前检查发现Fbg减低（0.4 g/L），剖宫产术后出血较多；2012年（29岁）发现“右心室占位及重度三尖瓣反流”，行“右室占位切除及三尖瓣成形术”，术后病理回报为“右心室血栓纤维化”，未抗凝治疗；2015年8月（32岁）因肺栓塞就诊于北京协和医院血液内科。先证者父母非近亲婚配，母亲无血栓史，父亲血栓史不详（已故），二伯有“肠系膜静脉血栓病史”，弟弟23岁时诊断为“肠系膜静脉血栓及双下肢深静脉血栓”。除先证者外，其余家系成员无出血症状。

2. 凝血指标检测：采用法国Stago STA-R全自动血凝仪通过凝固法测定凝血酶原时间（PT）、活化部分凝血活酶时间（APTT）和凝血酶时间（TT），检测试剂由德国Siemens公司提供。Fbg∶C采用Clauss法在法国Stago全自动血凝仪检测，Fbg∶Ag采用免疫比浊法检测。1∶1正浆纠正试验方法：将患者血浆与正混血浆（至少20例表观健康人混合血浆）1∶1混合后，37 °C孵育2 h，按上述方法检测PT、APTT、TT和Fbg∶C。

3. 纤维蛋白原聚集曲线：用pH 7.4的反应缓冲液A（50 mmol/L Tris-base、0.1mmol/L NaCl和2.5 mmol/L CaCl_2_）调节血浆Fbg浓度为0.5 g/L。将90 µl Fbg溶液（0.5 g/L）加入96孔平板，此后加入10 µl凝血酶（10 U/ml）。加入凝血酶后，立即用BIO-TEK微孔板分光光度计及KC4软件系统（美国BIO-TEK公司产品）在350 nm处进行连续动态读数，读数间隔为30 s，持续时间为60 min。

4. 测序平台及Panel选择：采用Illumina MiSeq平台进行pair-end测序，选取了与遗传性的出血性疾病、血小板疾病、血栓性疾病相关的126个基因，对转录本区域进行探针设计并合成（迈基诺），文库富集投入500 ng gDNA，平均测序深度300×。

5. 生物信息学分析及遗传学分析：FASTQ测序数据的生物信息学分析主要应用BWA-MEN 0.7.12进行参考序列比对，单核苷酸变异及插入缺失的分析使用GATK4.18 HaplotypeCaller，变异注释及过滤使用Annovar。

遗传学分析步骤如下：①数据质控：数据需满足对测序靶区域的覆盖大于95％，测序深度大于20×，常染色体杂合突变的突变频率应控制在0.4～0.6。②依据ACMG遗传变异分类标准指南对该突变进行遗传病分级。应用1000 Genomes、Genome Aggregation Database（GnomAD）和Exome Aggregation Consortium（ExAC）数据库对人群的次等位基因频率进行筛选，根据ACMG指南，设限为0.05[3]。③使用phyloP、phastCons程序利用隐马尔可夫模型分析突变位点对应氨基酸的保守性。通过SIFT、Polyphen2_HVAR、MutationTaster、LRT、FATHMM、CADD软件预测核苷酸变异对蛋白质结构及功能的影响。在UniProtKB 3D structure数据库中查询突变氨基酸所在功能域，通过Swiss-Pdb Viewer软件构建蛋白质模型。

## 结果

1. 先证者及家系成员凝血功能检测结果：先证者的凝血检查及1∶1正浆纠正试验结果如表1所示。先证者就诊我院前1月于当地医院诊断肺栓塞并加用华法林抗凝，故先证者凝血指标为应用华法林抗凝期间的检测结果，其PT、APTT、TT均明显延长，Fbg∶C明显减低。先证者血浆与正混血浆1∶1混合后TT可完全纠正，排除获得性抑制物的影响。先证者及家系成员的凝血指标如表2所示。先证者、弟弟及二伯均检测到Fbg∶C及Fbg∶Ag减低，Fbg∶C / Fbg∶Ag比值<0.7，提示为低异常纤维蛋白原血症。先证者母亲及儿子凝血指标无明显异常。

**表1:** 先证者凝血检查结果及1∶1正浆纠正试验

标本	APTT（s）	PT（s）	INR	TT（s）	Fbg∶C（g/L）
先证者血浆	71.1	45.0	3.66	28.0	0.60
正混血浆	26.0	11.4	0.97	18.4	2.79
先证者血浆∶正混血浆=1∶1	32.2	14.0	1.18	19.9	1.69

参考值	27.2~41.0	10.0~16.0		14.0~21.0	1.80~3.50

注：APTT：活化部分凝血活酶时间；PT：凝血酶原时间；INR：国际标准化比值；TT：凝血酶时间；Fbg∶C：纤维蛋白原活性

**表2:** 先证者及家系成员的凝血指标及基因检测结果

家系成员	APTT（s）	PT（s）	TT（s）	Fbg∶C（g/L）	Fbg∶Ag（g/L）	FGG基因c.1094G>A
先证者	71.1	45.0	28.0	0.60	1.0	杂合子
母亲	36.5	12.5	23.0	2.24	3.0	野生型
弟弟	51.8	19.8	28.4	0.56	0.8	杂合子
二伯	40.5	17.2	28.9	0.50	0.9	杂合子
儿子	26.7	12.6	28.9	0.50	0.9	野生型

参考值	27.2~41.0	10.0~16.0	14.0~21.0	1.80~3.50	2.0~4.0	

注：APTT：活化部分凝血活酶时间；PT：凝血酶原时间；TT：凝血酶时间；Fbg∶C：纤维蛋白原活性；Fbg∶Ag：纤维蛋白原抗原

2. 纤维蛋白原聚集曲线：纤维蛋白原聚集曲线示先证者、弟弟和二伯的纤维蛋白原无法聚集，而其母纤维蛋白原聚集曲线与正常人相近（图1）。因家系患病成员的纤维蛋白原无法聚集，故未行纤维蛋白溶解曲线。

**图1:**
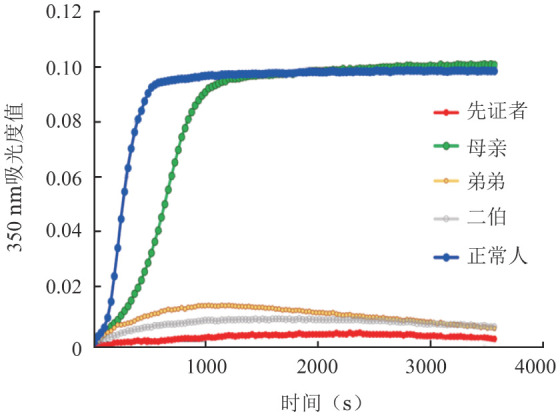
纤维蛋白原聚集曲线 先证者及其弟、二伯的纤维蛋白原无法聚集，而其母纤维蛋白原聚集曲线与正常人相近

3. 先证者及家系成员基因检测结果及遗传分析：先证者检出FGG:NM_000509.6: exon8: c.G1094A:p.C365Y杂合突变（图2），通过Sanger测序验证突变存在。其弟弟和二伯均检出相同位点杂合突变，其母亲和儿子为野生型（表2）。ACMG InterVar分析FGG：c.G1094A：p.C365Y突变的遗传病分级为PM1，为具有中等致病性证据。在ExAC、1000Genome和GnomAD数据库中均无该突变在人群中次等位基因的频率报道，提示该突变发生率极低。携带杂合突变的家系成员均有血栓病史且纤维蛋白原明显减低，提示该等位基因符合常染色显性的遗传方式。推测先证者的突变基因来自其父亲，因先证者就诊时其父已故，故父亲的基因型无法验证。

**图2:**
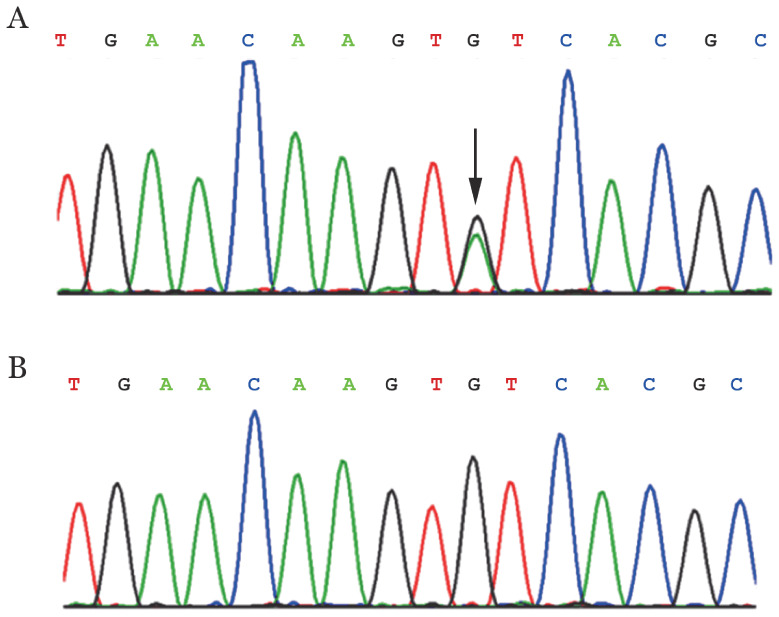
FGG基因c.1094G>A杂合突变（A）（箭头所示为突变位点）及野生型（B）

4. 氨基酸保守性分析：软件模型对该突变位点氨基酸的保守性分析如下：phyloP100way_ vertebrate（100种脊椎动物模型）评分为7.676分，phyloP30way_mammalian（30种哺乳动物模型）评分为1.026分，phastCons100way_vertebrate（100种脊椎动物模型）评分为1分，phastCons30way_mammalian（30种哺乳动物模型）评分为0.934分。以上模型计算FGG:p.C365氨基酸在同源物种间具有高度保守型。

5. 核苷酸变异对蛋白质结构及功能影响的预测分析：生物信息学软件对FGG：p.C365Y突变的预测结果如下：SIFT评分为0.004分，预测为有害；Polyphen2_HVAR评分为0.991分，预测为有害；MutationTaster评分为1分，预测为有害；LRT_pred预测为有害；FATHMM_pred预测为有害；CADD_phred评分为29.6分，大于10-20的常用阈值，有害性排名在CADD中接近前0.1％（30分）。以上软件分析均提示该突变对于蛋白质结构或功能存在较大影响，预测结果为有害突变。

通过UniProtKB 3D structure数据库查看FGG编码纤维蛋白原γ链的蛋白空间结构如图3A所示，其中Cys365如图3B所示。在空间结构上，Cys365与Cys352存在一个氢键连接（图3B中箭头所指黄色柱），与Asn363存在两个氢键连接（图3B中箭头所示蓝色虚线）。Cys365突变为Tyr365后（图3C），其与Cys352的氢键断裂，通过Swiss-Pdb Viewer预测Tyr365可能与Glu354形成新的氢键，导致蛋白质二级结构的改变。

**图3:**
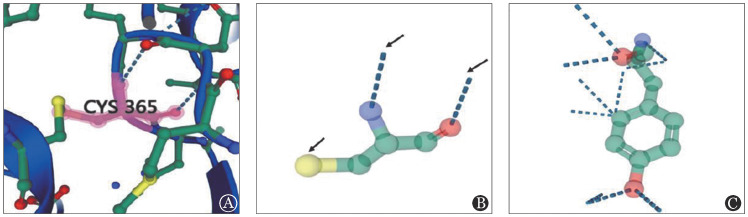
FGG基因编码纤维蛋白原γ链的蛋白质模型图 A：FGG编码纤维蛋白原γ链的蛋白空间结构；B：Cys365的氨基酸结构图；C：Tyr365的氨基酸结构图

## 讨论

Fbg是由Aα、Bβ和γ三对多肽链以二硫键连接而成的二聚体，编码基因分别为位于4号染色体4q31.3区域的FGA、FGB和FGG基因[1]。CD患者的基因缺陷类型以错义突变为主，其他的基因缺陷类型包括移码突变、小片段插入或缺失等[4]。低异常纤维蛋白原血症患者常见FGA和FGG基因突变，而无/低纤维蛋白原血症患者常见FGB基因突变[5]。欧洲的一项研究则显示，83.9％的致病突变发生在FGA基因的2号外显子和FGG基因的8号外显子[5]，与中国人群的研究结果[6]一致。此外，中国的家系报道、人群研究与欧洲的研究均提示两个热点突变分别为FGA Arg35和FGG Arg301[5]–[8]。以上两个热点突变均位于Fbg的重要功能区，FGA Arg35位于纤维蛋白原Aa链N端凝血酶剪切位点，FGG Arg301位于γ链端端对齐及纤维蛋白交联形成多聚体的位点。

本研究家系的患者为FGG:p.Cys365Tyr（c.1094G>A）杂合突变，该位点位于热点区域8号外显子。查阅Clinvar数据库未发现该位点突变的既往报道。http://site.geht.org/在线数据库记录了同一位点的错义突变，但替换的碱基和氨基酸类型均不同。该数据库记录的FGG基因突变位点分别为c.1094G>C，导致p.Cys365Ser，对应的表型为出血表现、无血栓表现，另一记录突变的氨基酸位点为p.Cys365Ser，对应表型同为轻度出血表现，均与本研究所报道的表型不同[9]–[10]。中国人群研究曾报道FGG基因相同氨基酸位点的突变（c.1093T>G，p.C365G），但碱基突变位点及氨基酸替换类型均与本家系不同。通过以上数据库检索及相关文献复习，初步确认本研究所报道家系的突变位点为国内外首次报道的新发突变。

CD患者的临床表现呈现明显异质性。Haverkate等[11]在1995年发表的一项纳入250例CD患者的回顾性研究显示，53％的患者无症状，26％表现为出血，以女性月经增多、皮肤黏膜出血、外伤或手术后出血为主，另21％表现为血栓，以静脉血栓最为常见，血栓及出血表现可发生于同一患者。后续几项较大规模的回顾性研究报道了更高的出血发生率（52％～63％）[12]–[14]。Casini等在2014年发表的回顾性研究纳入了101例CD患者，中位随访时间为8.8年，出血、血栓事件的千人年发生率分别为2.5、18.7，估算50岁时出血、血栓事件的累积发生率分别为19.2％、30.1％[14]。因既往多数研究的性质为横断面研究或荟萃分析，早期诊断的患者缺乏长期随访数据，对于出血事件缺乏标准化定义，发表偏倚对无症状携带者比例的低估，以上诸多原因导致各研究中所报道的出血及血栓事件的发生率存在较大差异且可能均存在一定偏倚。

本研究家系中患者的Fbg∶C及Fbg∶Ag水平均减低，Fbg∶C与Fbg∶Ag比值<0.7，提示异常纤维蛋白原血症与低纤维蛋白原血症共存，故诊断为遗传性低异常纤维蛋白原血症。可能的病理机制为基因缺陷同时影响到了纤维蛋白原的功能以及蛋白的组装和分泌，另有部分患者为复合杂合突变致病。与CD类似，遗传性低异常纤维蛋白原血症患者的基因型与表型同样存在明显的异质性。Casini等[2]的荟萃分析纳入了51例遗传性低异常纤维蛋白原血症的病例，其中22％的患者无症状，45％表现为轻度出血，主要为产科或妇科相关出血，43％至少发生过一次血栓事件。对比异常纤维蛋白原血症和低纤维蛋白原血症，低异常纤维蛋白原血症患者的血栓风险更高，且易出现早发血栓及复发血栓[2],[5]。本研究家系同样以血栓表型为主，先证者表现为反复血栓包括罕见部位的血栓形成，余患病家系成员均有血栓史。

由于CD等位基因的高度异质性，多数基因型与表型尚未建立明确的相关性，上述两个热点突变FGA Arg35和FGG Arg301与表型的关系亦未明确[14]–[15]。研究发现部分基因突变型与血栓表型存在相关性。如纤维蛋白原病CaracasⅤ，其突变位点为FGA:c.1595C>G，该突变影响了纤维蛋白与纤溶酶的结合位点，阻止了正常的纤溶过程[16]。其他的血栓相关突变型包括纤维蛋白原病Ijmuiden、New York Ⅰ、Nijmegen、Naples和Melun[17]–[21]。因此，对患者进行分子遗传学检测可能有助于判断患者的血栓风险。此外，体外功能性试验（如纤维蛋白原聚集曲线和纤维蛋白溶解曲线）以及血栓弹力图等实验室检测方法可能对预测患者的临床表型提供辅助信息[1]。本研究家系患病成员的纤维蛋白原聚集曲线提示纤维蛋白原聚集障碍，但家系成员出血表现不显著，体外功能试验对于表型的预测仍有待大规模研究进一步验证。

本研究应用生物信息学软件分析FGG p.Cys365Tyr突变的生物学特性。phyloPhe和phastCons软件分析发现FGG：p.Cys365在同源物种间具有高度保守性，提示该位点为纤维蛋白原分子的重要位点。多种在线生物信息学软件对FGG：p.Cys365Tyr突变分析均提示该突变对蛋白质结构或功能存在较大影响，预测结果为有害突变。Swiss-Pdb Viewer软件构建突变前后的蛋白模型显示该突变可能导致氢键连接位置及FGG蛋白空间结构的改变。通过上述生物信息学分析，进一步验证了本研究家系突变位点的致病性。

综上，本文报道了首例FGG基因c.1094G>A杂合突变所致遗传性低异常纤维蛋白原血症的家系，遗传方式为常染色体显性遗传，临床表型以血栓为主，伴轻度出血表现。通过体外功能性试验及生物信息学分析对其致病机制进行了初步探索。作为一种罕见的出凝血异常疾病，该疾病容易被漏诊或误诊，开展基因测序及相关功能学检查，可帮助患者明确诊断和协助预测临床表型。
